# Tuberculous Lymphadenopathy Mimicking Pancreatic Neoplasm

**DOI:** 10.1155/2012/579297

**Published:** 2012-07-19

**Authors:** Kunikazu Hoshino, Shingo Arakaki, Daisuke Shibata, Tatsuji Maeshiro, Akira Hokama, Fukunori Kinjo, Masayuki Shiraishi, Tadashi Nishimaki, Jiro Fujita

**Affiliations:** ^1^Department of Infectious, Respiratory, and Digestive Medicine, Faculty of Medicine, University of the Ryukyus, Nishihara, Okinawa 903-0215, Japan; ^2^Department of Endoscopy, University Hospital of the Ryukyus, Nishihara, Okinawa 903-0215, Japan; ^3^Division of Digestive and General Surgery, Faculty of Medicine, University of the Ryukyus, Nishihara, Okinawa 903-0215, Japan

## Abstract

Abdominal tuberculosis (TB) is the sixth most common location of extrapulmonary TB involvement. Because its symptoms and signs are often nonspecific, laboratory and imaging findings mimic other diseases including carcinoma. Therefore, the diagnosis of abdominal TB is challenging. We herein report a case of 74-year-old woman who presented with abdominal pain, anorexia, and weight loss. She had been given a diagnosis of pancreatic head carcinoma. Laboratory data was unremarkable except for elevated erythrocyte sedimentation rate, CA125, and sIL-2R. CT scan revealed multiple enlarged peripancreatic lymph nodes and concentric thickening of the ileocecal wall. Colonoscopy demonstrated deformed ileocecal valve and erosions. Histological examination showed epithelioid granulomas. Laparoscopy revealed numerous white tubercles diffusely covering the parietal peritoneum. Histopathological images of peripancreatic lymph node revealed large multiple caseating granulomas surrounded by Langhans_giant cells and epithelioid cells. Polymerase chain reaction and culture of the specimens were positive for *Mycobacterium tuberculosis*. Tuberculous lymphadenopathy, colitis, and peritonitis were finally diagnosed. She responded well to the antitubercular treatment.

## 1. Introduction

Abdominal tuberculosis (TB) is the sixth most common location of extrapulmonary TB. It can involve any abdominal organ. Because its symptoms and signs are often insidious and nonspecific, diagnosis of abdominal TB sometimes accompanies with great difficulties. We herein report a case of tuberculous peripancreatic lymphadenopathy mimicking pancreatic neoplasm. Importance of various investigations for definitive diagnosis is discussed.

## 2. Case Presentation

**Figure 1:**
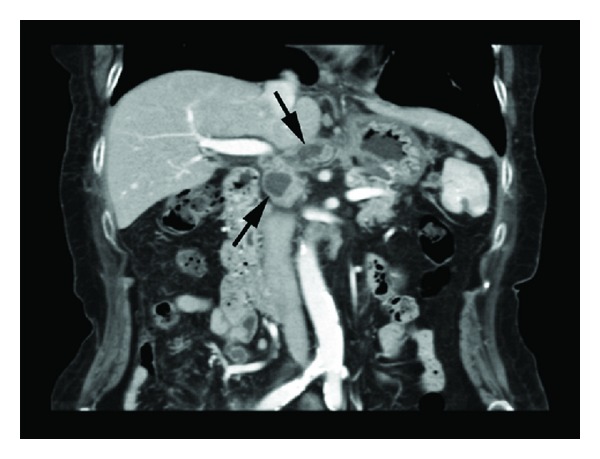
Abdominal computed tomography scan revealed multiple enlarged lymph nodes (arrows).

**Figure 2:**
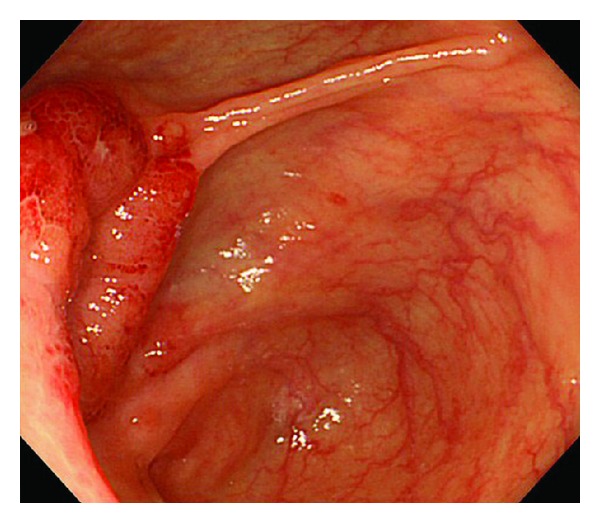
Endoscopic view of deformed and edematous ileocecal valve with erythema.

**Figure 3:**
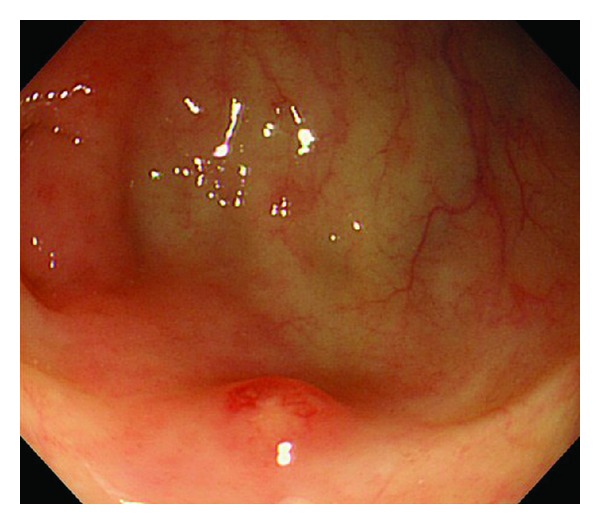
A tiny erosive nodule near the appendiceal orifice.

**Figure 4:**
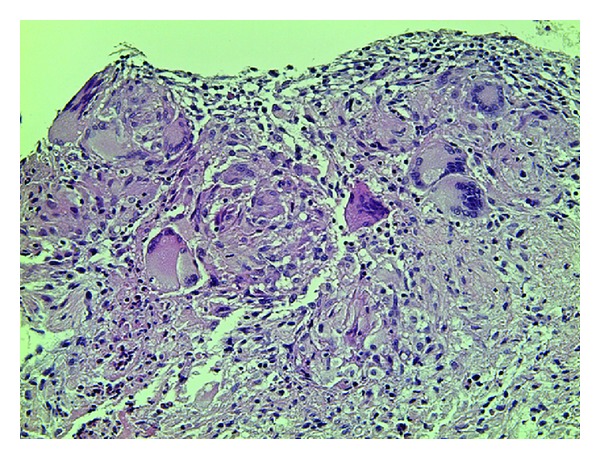
Histopathological image of the nodule (Figure 3) showing epithelioid granulomas with multinucleated giant cells (hematoxylin and eosin staining, ×200).

A 74-year-old woman presented initially to a hospital with a 3-week history of abdominal pain, anorexia, and weight loss of 5 kg over 3 months. As abdominal computed tomography (CT) scan showed a cystic lesion in the pancreatic head, she was referred to our university hospital with the diagnosis of pancreatic head carcinoma. She denied fever, chills, nausea, vomiting, and diarrhea. On admission she was afebrile. On physical examination she had mild tenderness in the right lower quadrant. Her medical history was unremarkable. She had an uncle who had been treated for TB. Laboratory tests showed an elevated erythrocyte sedimentation rate (111 mm/hr, 2 < normal value < 16 mm/hr), serum CA125 level (83 U/mL, normal value < 35 U/mL), and serum sIL-2R level (1706 U/mL, 127 < normal value < 582 U/mL). She had a positive tuberculin skin test and a positive QuantiFERON-TB-2 Gold (Cellestis) result. Though chest X-ray was unremarkable, chest CT scan revealed a swollen hilar lymph node (8 mm). Abdominal CT scan showed multiple enlarged peripancreatic lymph nodes (arrows), concentric thickening of the ileocecal wall, and several segmental lesions of the ileum (Figure 1), but pancreatic mass was absent. Esophagogastroduodenoscopy was unremarkable. Colonoscopy demonstrated deformed and edematous ileocecal valve with erythema (Figure 2) and a tiny erosive nodule near the appendiceal orifice (Figure 3). Histopathological examination of the nodule disclosed epithelioid granulomas (Figure 4). Though polymerase chain reaction (PCR) of the biopsied specimen was negative, culture was positive for *Mycobacterium tuberculosis*.

**Figure 5:**
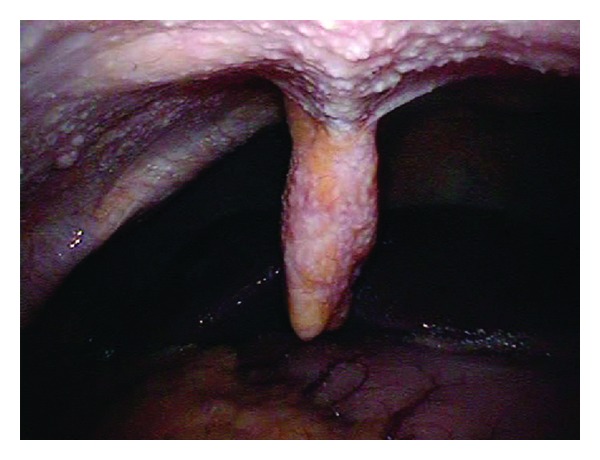
Laparoscopic view demonstrating numerous white tubercles diffusely covering the parietal peritoneum, suggesting tuberculous peritonitis.

**Figure 6:**
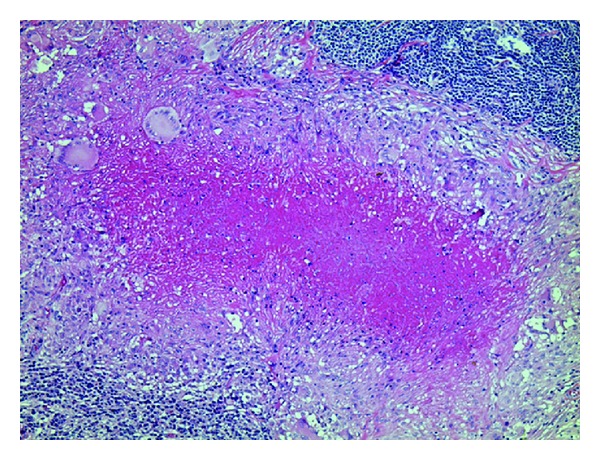
Histopathological image of the dissected abdominal lymph node disclosed multiple caseating granulomas with inflammatory cells including Langhans giant cells (hematoxylin and eosin staining, ×100).

Laparoscopic biopsy of the abdominal lymph nodes was performed to exclude other diseases, including pancreatic neoplasm and malignant lymphoma. Laparoscopy revealed numerous white tubercles diffusely covering the parietal peritoneum (Figure 5). Biopsies from the ligamentum teres hepatis and peripancreatic enlarged lymph nodes were obtained. Histopathological images of the former specimen revealed multiple epithelioid granulomas around fatty tissue. The latter contained liquefied necrotic material, and histopathological examination demonstrated large multiple caseating granulomas surrounded by Langhans' giant cells and epithelioid cells with infiltration of inflammatory cells (Figure 6). PCR and culture of the specimen were positive for *M. tuberculosis*. The organism was susceptible to isoniazid, rifampin, pyrazinamide, and ethambutol. On the basis of these findings, tuberculous lymphadenopathy, colitis, and peritonitis were finally diagnosed. Her symptoms improved after a few days of a four-drug regimen of antitubercular treatment. She remains well after six months of the treatment.

## 3. Discussion

Abdominal TB is the sixth most common location of extrapulmonary involvement [1]. It can occur at any part of abdominal organ, including the gastrointestinal tract, solid viscera, peritoneum, and abdominal lymph nodes [2]. The symptoms and signs are often nonspecific, laboratory and imaging findings may mimic other diseases including carcinoma. Therefore, the diagnosis of abdominal TB sometimes accompanies with great difficulties. The tuberculin skin test (TST) has been performed as a standard immunologic test for TB infection. However, because of false-positive results caused by the bacillus Calmette-Guerin (BCG) vaccine and nontuberculous mycobacteria infection, the interpretation of TST results is problematic [3]. Interferon-*γ* release assays (IGRAs), the QuantiFERON-TB-2 Gold (Cellestis), and T-SPOT.TB (Oxford Immunotec) are noninvasive immunologic diagnostic tests and measure T-cell-induced IFN-*γ* responses to early secretory antigenic target 6-kD (ESAT-6) protein and culture filtrate protein 10 (CFP-10) as *M. Tuberculosis*-specific antigens. QuantiFERON-TB-Gold In-Tube (Cellestis) has also been available, of which antigens include ESAT-6, CFP-10, and TB 7.7. Although the diagnostic value for the active TB infection in low- and middle-income countries is still unclear [4], IGRAs demonstrate superior sensitivity and specificity for active TB infection than TST [5, 6].

In abdominal TB, lymphadenopathy is a common manifestation. The mesenteric, celiac, porta hepatis, and peripancreatic lymph nodes are characteristically involved. Contrast-enhanced CT scan typically shows peripherally enhancing lymph nodes with central hypodensity, reflecting peripheral active inflammation with central caseation [1, 7]. When peripancreatic lymph nodes are remarkably conspicuous, they might be misdiagnosed as masses in the pancreas [8–10]. Differential diagnosis of pancreatic mass lesions and/or peripancreatic lymphadenopathy includes many diseases, such as inflammatory pseudotumor due to chronic pancreatitis, malignant neoplasm, malignant lymphoma, Whipple's disease, and infection with *M. avium-intracellulare* [7]. For the definitive diagnosis of malignancy, tuberculous disorders, or anther possible disease, histological and bacteriological examination is necessary. To obtain adequate samples, invasive procedures should be considered. Endoscopic ultrasound-guided fine-needle aspiration (EUS-FNA) is a safe, well-established technique, which is useful for extrapulmonary TB including peripancreatic lymph nodes [10–12]. Though, this method highly depends on the experience of operator, its clinical availability remains relatively limited. Percutaneous (US/CT-guided) biopsy is also useful technique, but small or deep lesions are difficult to sample, false-negative value of percutaneous FNA has been reported around 58% [13], which is also problematic. Laparoscopic biopsy is an alternative choice for abdominal lymphadenopathy. This technique, a less invasive procedure than a laparotomy, can approach deep lymphatic tissue and obtain the entire lymph nodes [14, 15]. To avoid inappropriate invasive procedures, preoperative definitive diagnosis should be established as much as possible [9].

Because of the existence of large aggregated lymphoid tissue, the ileocecal area and jejunoileum are the most common involvement of gastrointestinal tuberculosis. Colonoscopic findings of intestinal TB include nodules, ulcers, strictures, pseudopolyps, and deformed ileocecal valve [1, 2]. Pathologically, the submucosa and serosa are the most active inflammation sites and tuberculous granulomas are known to be located in the deep submucosa [16]. Therefore, multiple deep biopsies should be taken from the edge of ulcers or erosions, which suggest active lesions, for a histopathological or bacteriological diagnosis [2, 17]. Though Ziehl-Neelsen's staining for acid-fast bacilli or culture of the biopsied specimens is the gold standard to diagnose TB, the sensitivity of these tests is relatively low. On histopathological examination, granulomas were seen only in 8–48% of cases and caseations were even less seen (0–38% of positive cases) [2, 18]. Though the combination of culture and histopathology makes diagnostic yield a little higher, it is reported to be 30–80% [1]. PCR is a newer method, which has a better sensitivity (64–75%) and specificity (68–100%). PCR of the biopsied specimens is a useful technique and is recommended in addition to the conventional methods [1, 19].

Tuberculous peritonitis is one of the most common locations of abdominal TB [20]. Three forms have been reported: the wet type, the fibrotic-fixed type, and the dry type. Ascites is characteristically yellow citrine, exudative lymphocytic fluid, which has high density (25–45 HU) on CT scan [7, 21]. Though isolation of mycobacteria by culture leads to definitive diagnosis, the sensitivity of solid culture medium is approximately 35%. Laparoscopy is a safe, diagnostic choice for tuberculous peritonitis. Typically, laparoscopic examination reveals, as in our case, regular white equivalent-sized tubercles diffusely distributed on the peritoneum, whose findings have an excellent diagnostic value [2, 21]. Jazet et al. reported two cases of retroperitoneal tuberculosis with rare manifestations including obstructive jaundice and hematemesis [22].

Treatment of abdominal TB is basically as the same as that of pulmonary involvement. The currently recommended protocol is a 6-month course including isoniazid, rifampin, pyrazinamide, and ethambutol daily given for 2 months followed by isoniazid and rifampin for 4 months. Approximately 90% of patients with abdominal TB respond well to medical therapy [20]. The emergence of multidrug-resistant TB (MDR TB) and extensively drug-resistant TB (XDR TB) has been increasing during the past decade [23]. In Japan, the prevalence of MDR TB and XDR TB was reported to be 1.9% and 0.5%, respectively [24]. According to the analysis of 55 MDR/XDR cases in Japan by Murase et al., 17 (31%) of all the MDR cases were XDR, 30 cases (45%) of MDR/XDR cases were previously treated, and the odds ratio of previous treatment was 10.55 [25]. The susceptibility test should be performed before or during the treatment especially in the high-prevalence area.

In conclusion, the clinical presentation of abdominal TB is insidious and nonspecific, the diagnosis is frequently delayed. In patients with nonspecific abdominal symptoms, abdomen tuberculosis should be considered for prompt diagnosis and early start of the antitubercular treatment. In order to avoid misdiagnosis of a potentially fatal yet curable disease as an untreatable malignancy, various investigations, including laboratory tests, radiologic images, endoscopy, laparoscopy, histopathological, and bacteriological examination, should be performed for definitive diagnosis.
